# Supplementary material for: Preparation, Properties and Water Dissolution Behavior of Polyethylene Oxide Mats Prepared by Solution Blow Spinning
Source: Polymers (Basel). 2022 Mar 23;14(7):1299. doi: 10.3390/polym14071299 (PMC9003185; doi:10.3390/polym14071299)
Supplement: Supplementary file 1 [file polymers-14-01299-s001.zip › polymers-1634347-supplementary.pdf]

Supplementary Material

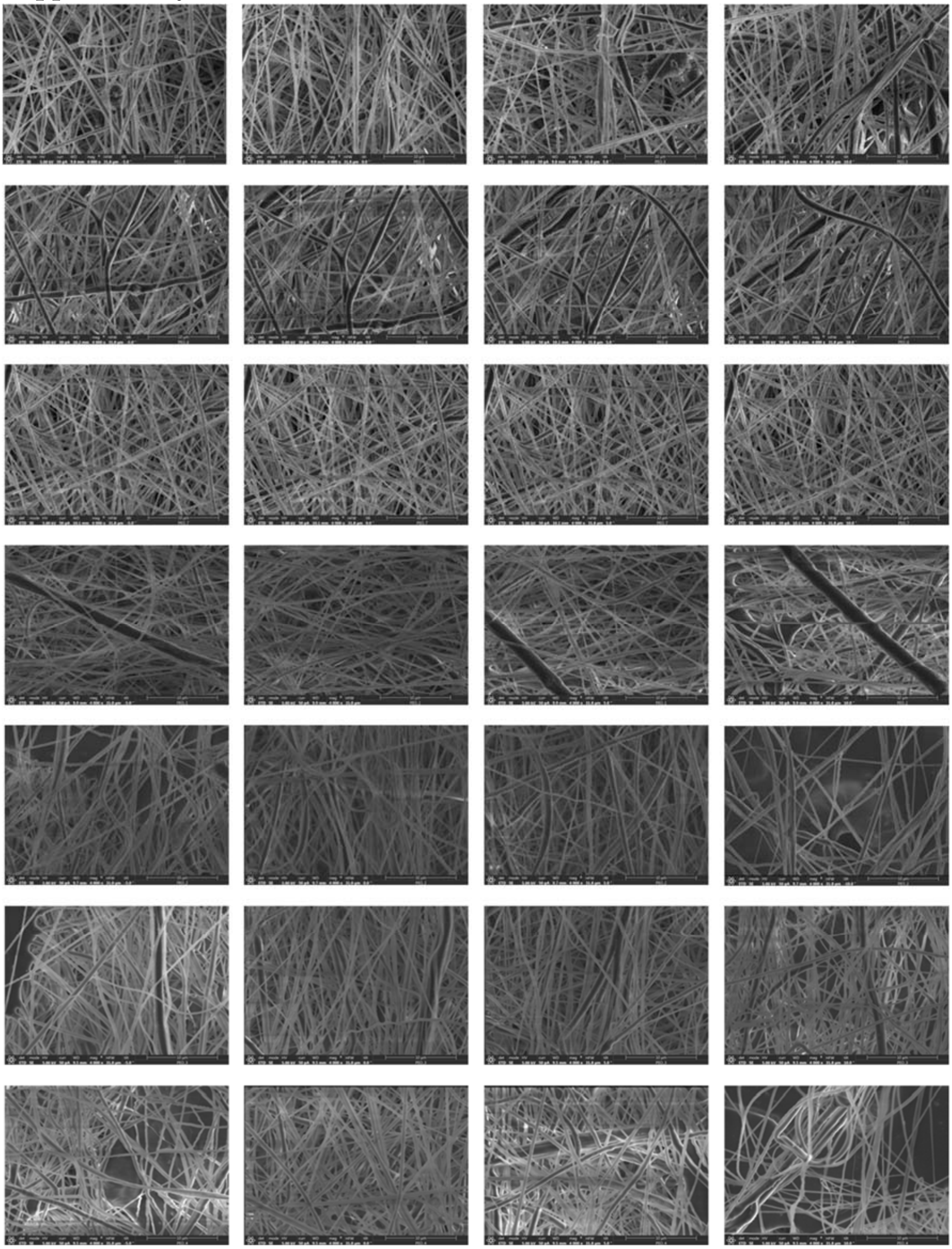

Figure S1. *SEM images of the different PEO developed materials with a 4000x magnification at different tilts. Columns (from left to right) represent the different tilt angles: -5, 0, 5 and 10 degrees; whereas rows (from top to button) represent the different materials: PEO-46, PEO-55, PEO-64, PEO-73, PEO-82, PEO-91AND PEO-100.*

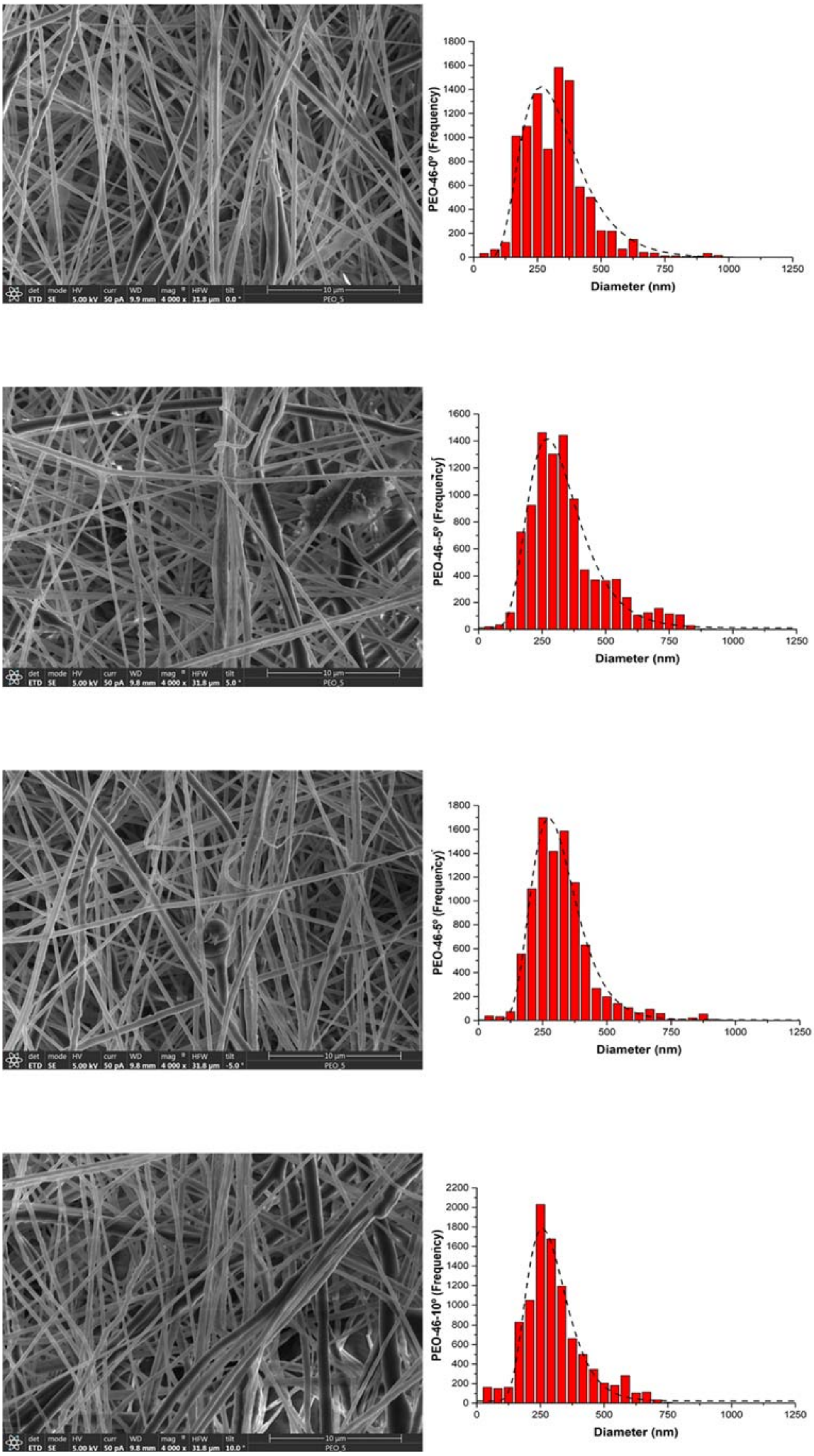

Figure S2. *Fiber size distributions of PEO-46 as a function of the tilt.*

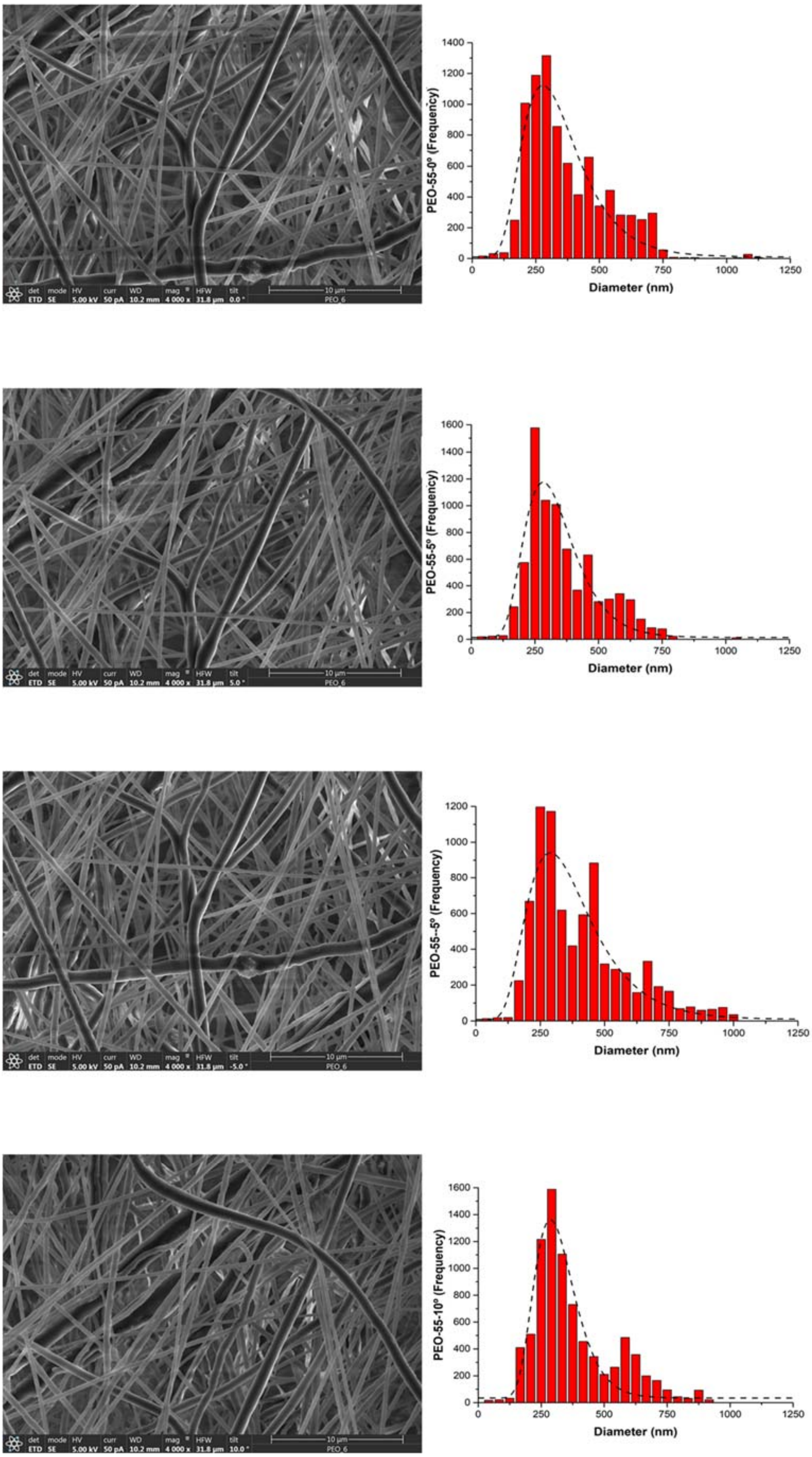

Figure S3. *Fiber size distributions of PEO-55 as a function of the tilt.*

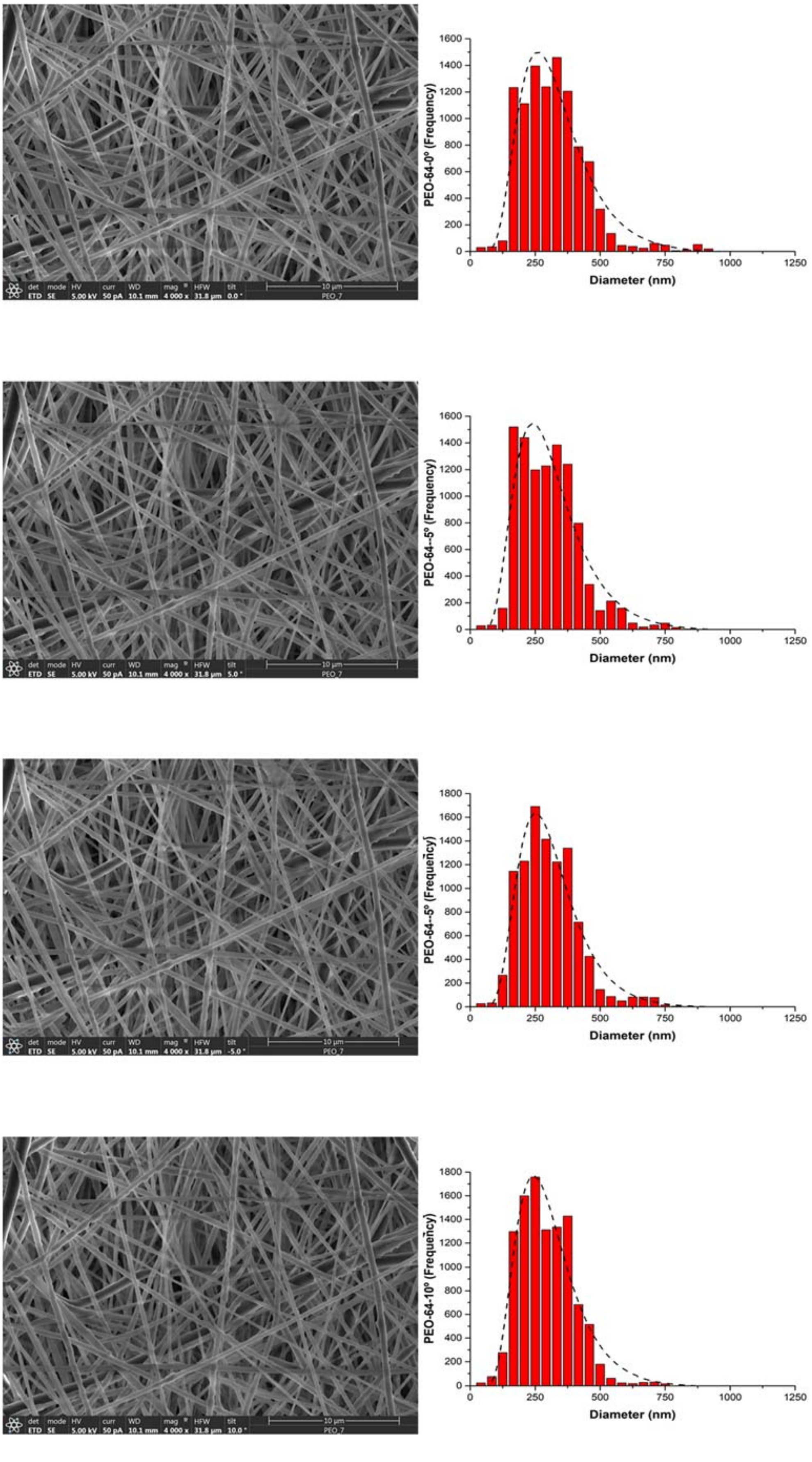

Figure S4. *Fiber size distributions of PEO-64 as a function of the tilt.*

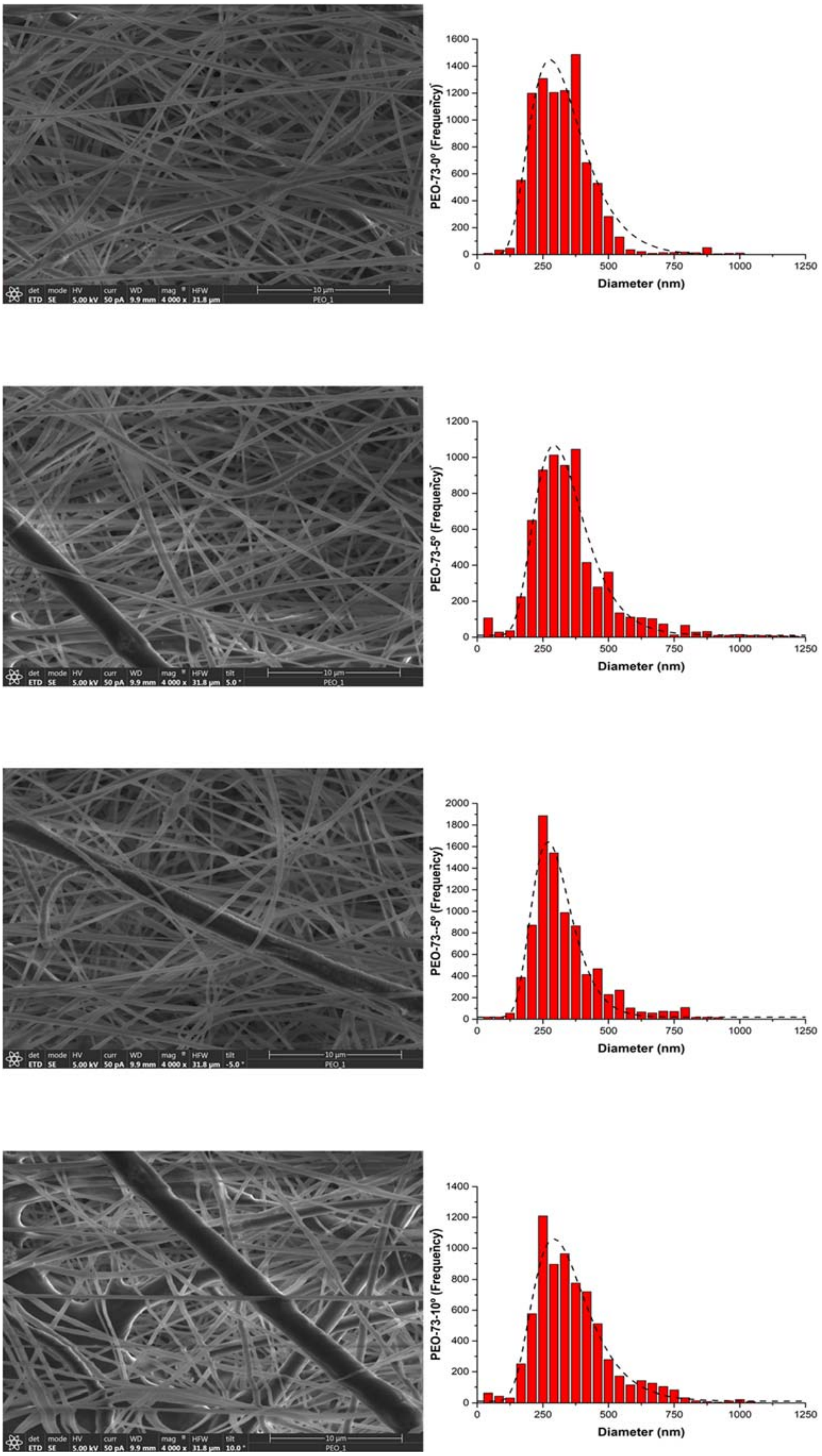

Figure S5. *Fiber size distributions of PEO-73 as a function of the tilt.*

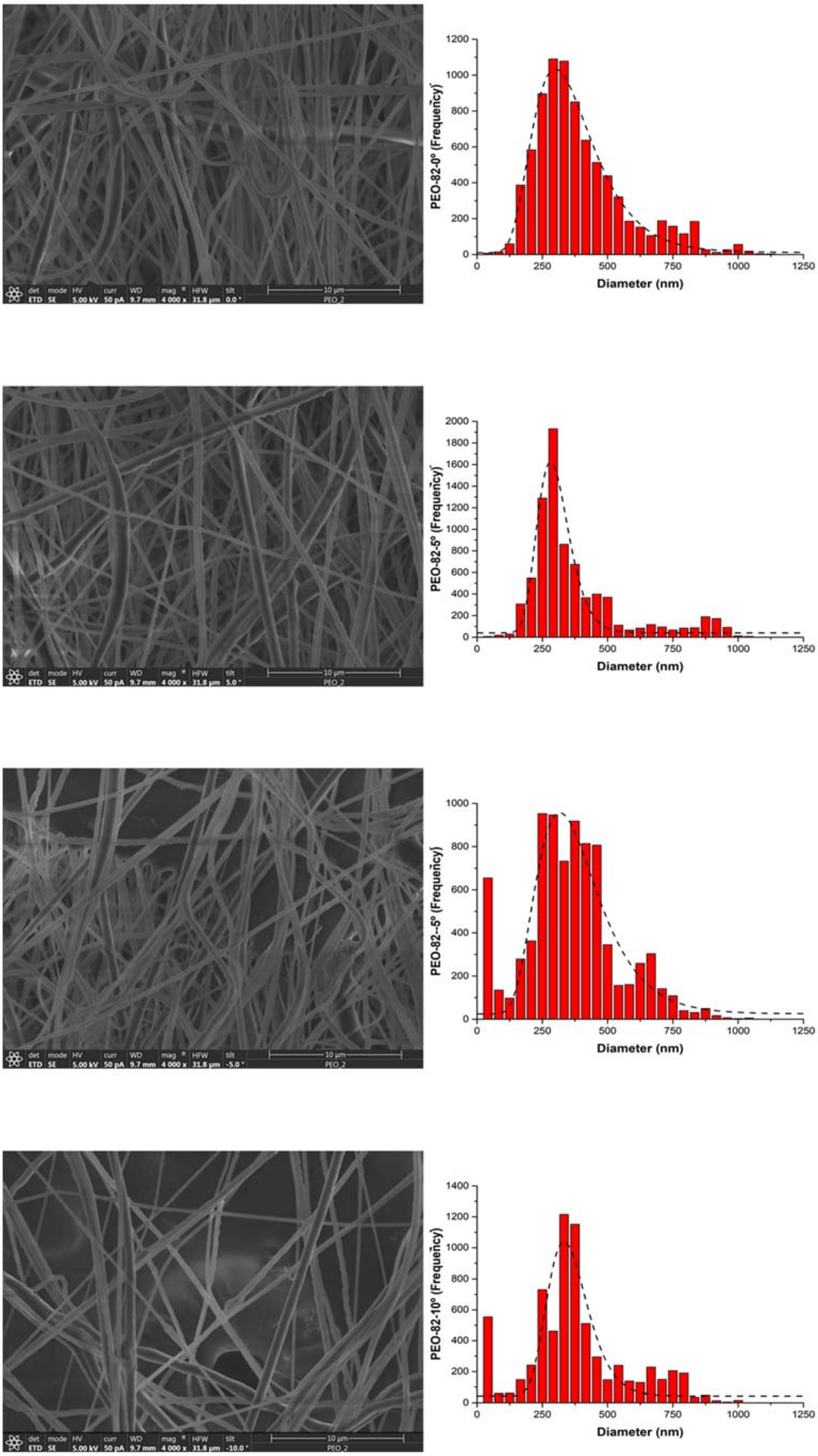

Figure S6. *Fiber size distributions of PEO-82 as a function of the tilt.*

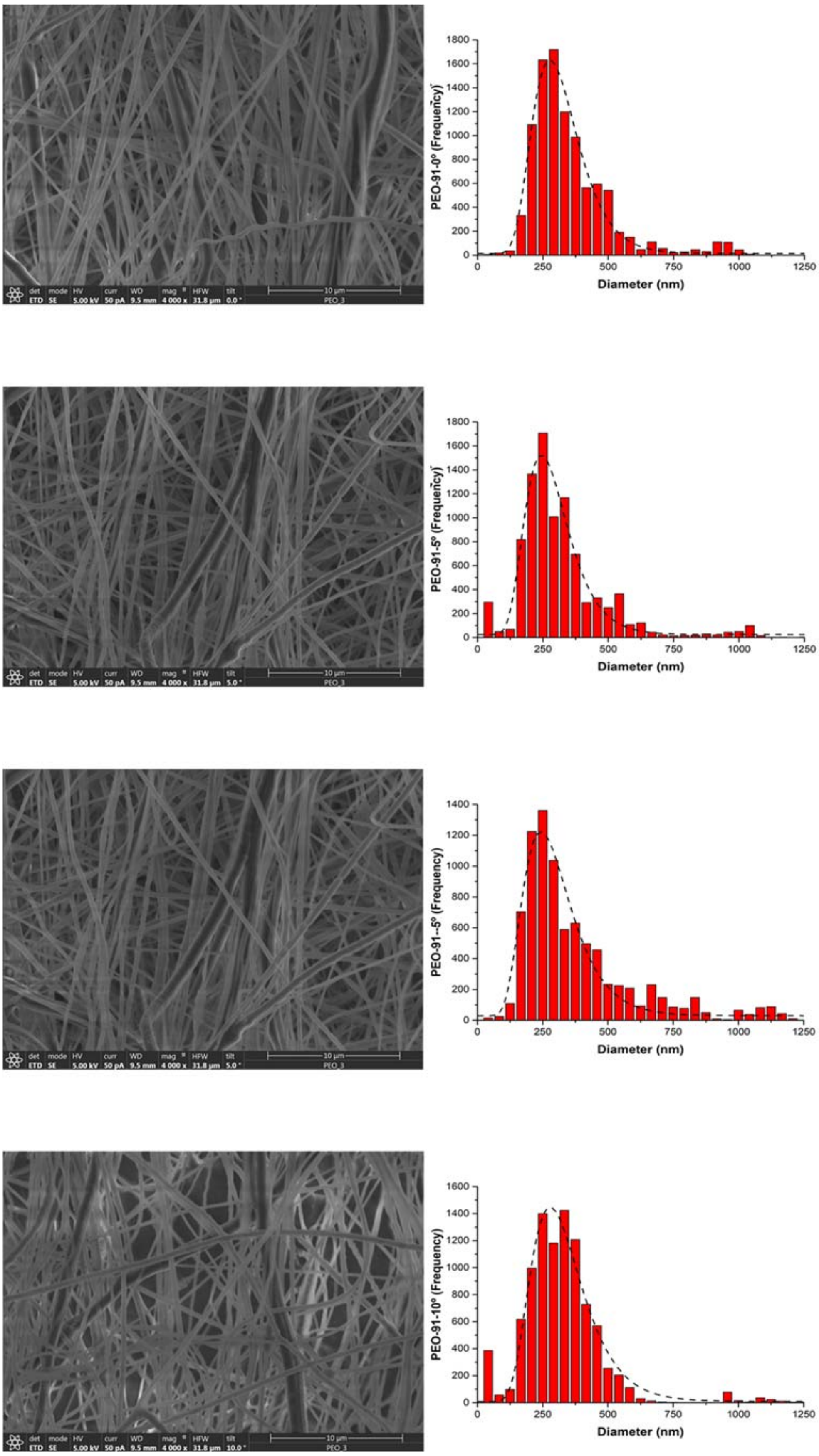

Figure S7. *Fiber size distributions of PEO-91 as a function of the tilt.*

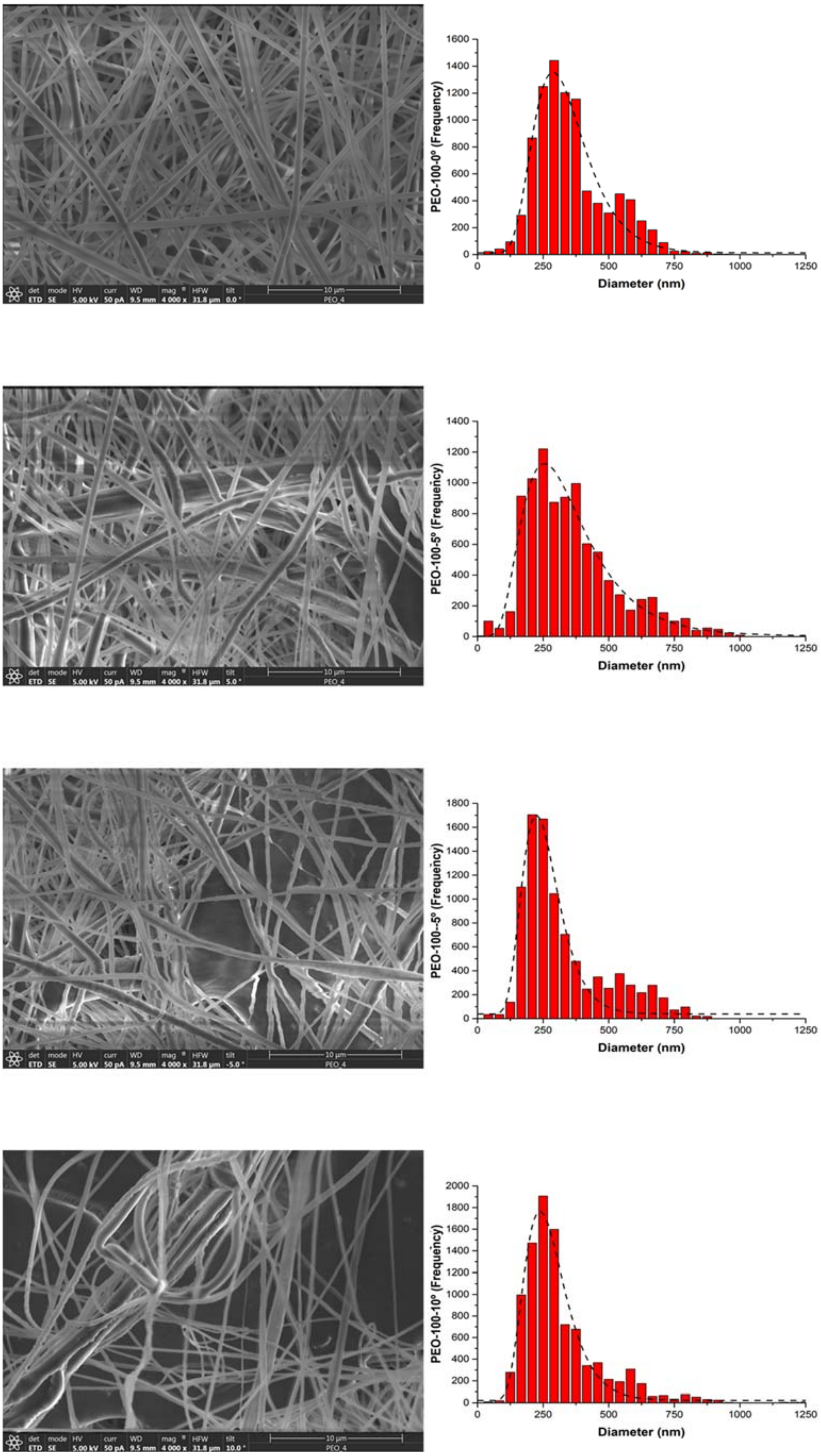

Figure S8. *Fiber size distributions of PEO-100 as a function of the tilt.*

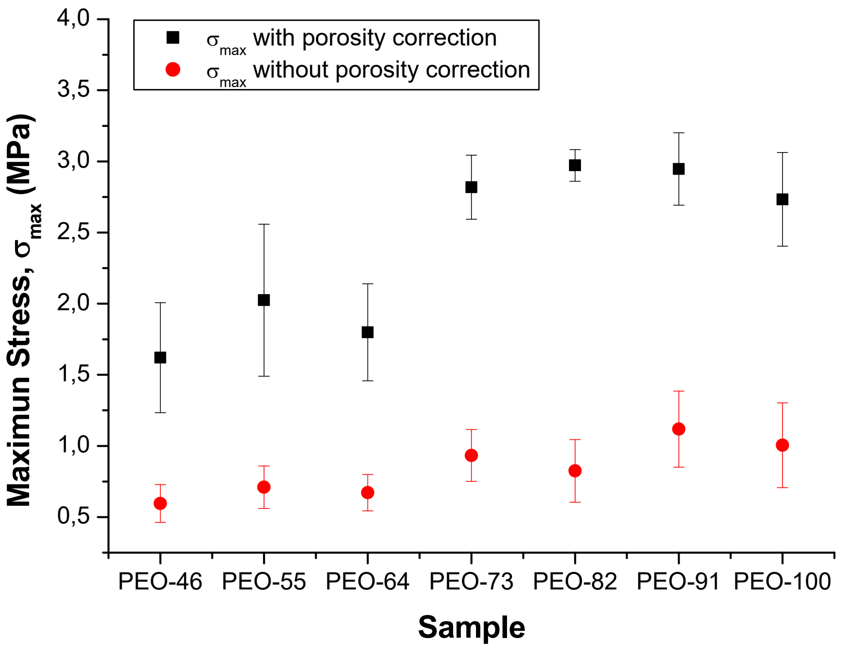

Figure S9. *Maximum stress as a function of PEO based material.*

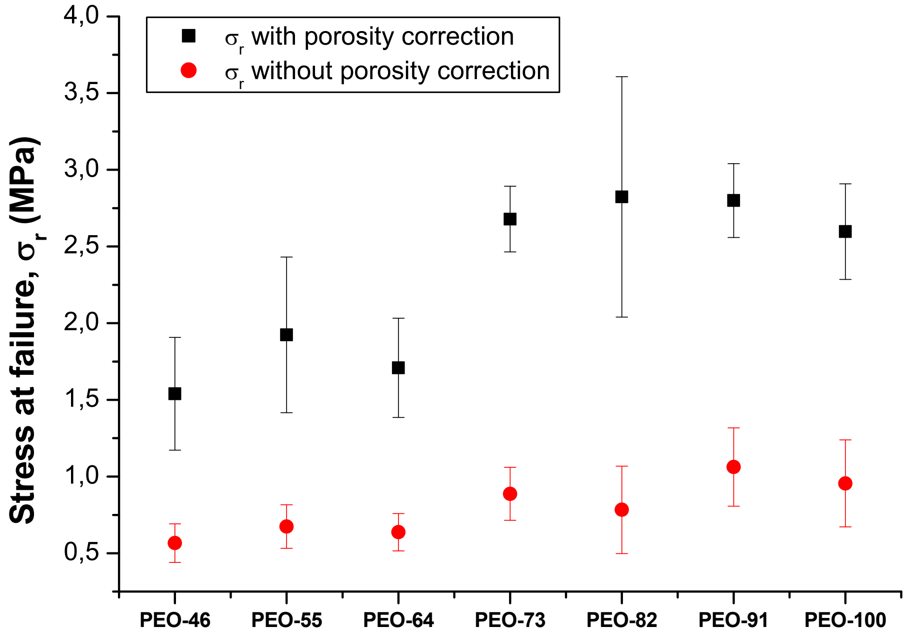

Figure S10. *Stress at failure as a function of PEO based material.*

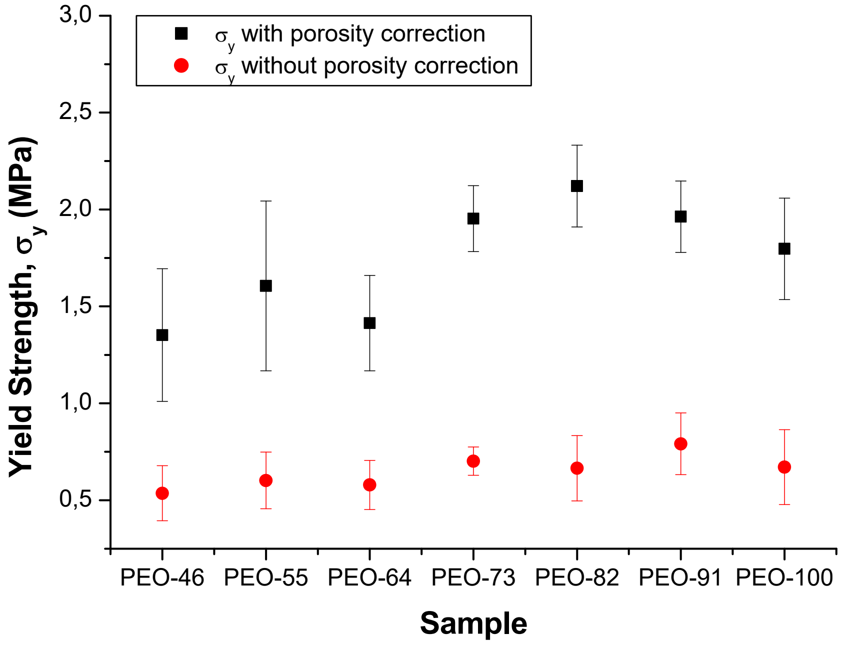

Figure S11. *Yield stress as a function of PEO based material.*
